# *Gardenia Jasminoides* extract is neuroprotective in an *in vitro* Tauopathy model

**DOI:** 10.1002/alz70855_097197

**Published:** 2025-12-23

**Authors:** Inas Birekdar

**Affiliations:** ^1^ The American University in Cairo, Cairo, Cairo, Egypt

## Abstract

**Background:**

Alzheimer's disease (AD) is a neurodegenerative disorder characterized by progressive loss of neuronal cells in different brain's regions which leads to cognitive impairment. The disease is characterized by Neurofibrillary tangle (NFT) or the abnormal accumulation of hyper phosphorylated Tau protein inside the neuronal cells. Effective treatment for neurodegenerative diseases (NDs) presents a clinical challenge. Neuroprotection is a promising strategy to prevent disease progression and enhance cognitive function.

**Objectives:**

In the current study we aim to assess Gardenia Jasminoides extract which contains Geniposide, a known biologically active and a neuroprotective agent, in preventing Tauopathy in an in vitro model of neurotoxicity using Zinc sulfate on human neuroblastoma SH‐SY5Y cells.

**Method:**

Zinc sulfate (100 μM, for 4 hours) was used on neuroblastoma SH‐SY5Y cells to establish an *in vitro* Neurotoxicity as well as Tauopathy model. Pretreatment with Gardenia Jasminoides extract (100 μM, for 2 hours) showed significant neuroprotective activity. Gardenia Jasminoides extract effectively reversed the cytotoxic effect of Zinc sulfate neuroblastoma SH‐SY5Y cells and significantly enhanced the cell viability.

**Result:**

Zinc sulfate (100 μM, for 4 hours) was used on neuroblastoma SH‐SY5Y cells to establish an *in vitro* Neurotoxicity as well as Tauopathy model. Pretreatment with Gardenia Jasminoides extract (100 μM, for 2 hours) showed significant neuroprotective activity. Gardenia Jasminoides extract effectively reversed the cytotoxic effect of Zinc sulfate neuroblastoma SH‐SY5Y cells and significantly enhanced the cell viability. On going work is aiming to investigate the ability of Gardenia Jasminoides extract to decrease the hyper‐phosphorylated Tau protein, as well as reveal the molecular pathway by which Gardenia Jasminoides extract works, by studying different protein kinases such as (PI3K, AKT, mTOR, *p*‐GSK‐3β, and *p*‐Tau).

**Conclusion:**

Gardenia Jasminoides extract shows promising neuroprotective potential in an *in vitro* model of Zinc induced Neurotoxicity as well as Tauopathy.

